# Cardiovascular Risk Assessment and Monitoring in Dementia Patients Prescribed Antipsychotics: A Multi-Centre Audit

**DOI:** 10.1192/bjo.2026.11737

**Published:** 2026-06-30

**Authors:** Celine Chan Ah Song, James Finlay

**Affiliations:** Northern Ireland Medical and Dental Training Agency, Belfast, United Kingdom

## Abstract

**Aims:**

Antipsychotics are frequently prescribed for behavioural and psychological symptoms of dementia (BPSD), despite well-established associations with increased cardiovascular morbidity and mortality. National and international guidelines recommend careful baseline cardiovascular assessment, ECG monitoring, metabolic screening, and documentation of risk–benefit discussions when initiating antipsychotics in older adults. Patients with dementiarepresent a particularly vulnerable group, often with multiple cardiovascular risk factors, yet adherence to monitoring standards remains variable.

The aim of this audit is to evaluate compliance with recommended cardiovascular risk assessment, monitoring, and documentation standards in dementia patients prescribed antipsychotics across inpatient mental health services.

**Methods:**

A retrospective audit was conducted across three wards in two hospitals. Twenty inpatients with a diagnosis of dementia who were prescribed antipsychotics for BPSD were included. Data was extracted from electronic medical records, focusing on baseline cardiovascular history, physical observations, ECG monitoring, metabolic investigations, documentation of QTc intervals, ongoing monitoring, medication review, and evidence of documented cardiovascular risk discussions. Standards were derived from NICE guidance and local trust policies.

**Results:**

The mean age at admission was 77.3 years, with an average length of stay of 192.6 days. All patients were detained under the Mental Health Order (Northern Ireland). Antipsychotics prescribed included risperidone (n=9), olanzapine (n=5), quetiapine (n=4), and haloperidol (n=2). Six patients had a documented cardiac history, though one was not recorded on admission. Hypertension was present in eight patients, diabetes in three, and atrial fibrillation in three.

Baseline physical observations were largely completed, with blood pressure, heart rate, and weight recorded in 19 of 20 patients. However, ECG monitoring was inconsistent: only eight patients had an ECG on admission, and just three had an ECG performed before or within 72 hours of antipsychotic initiation. QTc was documented in six patients, with appropriate action taken in the single case of prolongation. Metabolic monitoring was incomplete, with HbA1c checked in only 11 patients and ongoing metabolic monitoring documented in seven. While medication was reviewed regularly in MDT meetings for all patients, there was no documentation in any case of cardiovascular risks or risk–benefit discussions relating to antipsychotic use.

**Conclusion:**

This audit demonstrates significant gaps in cardiovascular risk assessment and documentation of risk–benefit discussions in dementia patients prescribed antipsychotics. Although routine observations and medication reviews were consistently performed, adherence to guideline-recommended cardiovascular monitoring was poor. Future targeted interventions could include increasing awareness amongst staff, standardized prescribing checklists, and electronic prompts, are required to improve patient safety and ensure compliance with best practice.

